# Principles of fluid management and stewardship in septic shock: it is time to consider the four D’s and the four phases of fluid therapy

**DOI:** 10.1186/s13613-018-0402-x

**Published:** 2018-05-22

**Authors:** Manu L. N. G. Malbrain, Niels Van Regenmortel, Bernd Saugel, Brecht De Tavernier, Pieter-Jan Van Gaal, Olivier Joannes-Boyau, Jean-Louis Teboul, Todd W. Rice, Monty Mythen, Xavier Monnet

**Affiliations:** 1 Intensive Care Unit, University Hospital Brussels (UZB), Laarbeeklaan 101, 1090 Jette, Belgium; 2 Faculteit Geneeskunde en Farmacie, Vrije Universiteit Brussel (VUB), Brussels, Belgium; 3 Intensive Care Unit, ZiekenhuisNetwerk Antwerpen, ZNA Stuivenberg, Lange Beeldekensstraat 267, 2060 Antwerpen 6, Belgium; 4 Department of Anesthesiology, Centre of Anesthesiology and Intensive Care Medicine, University Medical Centre Hamburg-Eppendorf, Hamburg, Germany; 5 Service d’Anesthésie-Réanimation 2, CHU Bordeaux, 33000 Bordeaux, France; 6 Medical Intensive Care Unit, Hopitaux universitaires Paris-Sud, AP-HP, Université Paris-Sud, Le Kremlin-Bicetre, France; 7 University College London Hospitals, National Institute of Health Research Biomedical Research Centre, London, UK; 8 Division of Allergy, Pulmonary and Critical Care Medicine, Vanderbilt University School of Medicine, Nashville, TN USA

**Keywords:** Fluids, Fluid therapy, Fluid management, Fluid stewardship, Four D’s, Four indications, Four hits, Four phases, Four questions, Resuscitation, Antibiotics, Drug, Dose, Duration, De-escalation, De-resuscitation, Maintenance, Replacement, Goal-directed therapy, Monitoring, Fluid responsiveness, Passive leg raising

## Abstract

In patients with septic shock, the administration of fluids during initial hemodynamic resuscitation remains a major therapeutic challenge. We are faced with many open questions regarding the type, dose and timing of intravenous fluid administration. There are only four major indications for intravenous fluid administration: aside from resuscitation, intravenous fluids have many other uses including maintenance and replacement of total body water and electrolytes, as carriers for medications and for parenteral nutrition. In this paradigm-shifting review, we discuss different fluid management strategies including early adequate goal-directed fluid management, late conservative fluid management and late goal-directed fluid removal. In addition, we expand on the concept of the “four D’s” of fluid therapy, namely drug, dosing, duration and de-escalation. During the treatment of patients with septic shock, four phases of fluid therapy should be considered in order to provide answers to four basic questions. These four phases are the resuscitation phase, the optimization phase, the stabilization phase and the evacuation phase. The four questions are “When to start intravenous fluids?”, “When to stop intravenous fluids?”, “When to start de-resuscitation or active fluid removal?” and finally “When to stop de-resuscitation?” In analogy to the way we handle antibiotics in critically ill patients, it is time for fluid stewardship.

## Background

In patients with septic shock, hemodynamic stabilization using intravenous fluids remains a major therapeutic challenge as numerous questions remain regarding the type, dose and timing of fluid administration. In these patients, fluids play an important role beyond hemodynamic stabilization and resuscitation. Intravenous fluids should be prescribed as any other drug we give to our patients: we should take into account the indications and contraindications for different types of fluids [2–8]. We should only prescribe fluids when they are clearly indicated and should balance the risk of not administering enough with the increasingly apparent risks of too much fluid.

In this review, we will expand on the concept of the “four D’s” of fluid therapy (drug, duration, dosing and de-escalation). We will also focus on the recent concept defining four different phases in the time course of septic shock (resuscitation, optimization, stabilization and evacuation). Each phase requires a different therapeutic attitude regarding fluid administration. Taking into account both of these concepts in combination with other suggested ideas may promote more rational fluid administration aimed at avoiding both too little and too much. In analogy to the way we handle antibiotic usage in the critically ill, it is now time for fluid stewardship.

## The risk of fluid overload

Treating a patient with septic shock inevitably results in some degree of salt and water overload. First and foremost, this is the result of the initial fluid resuscitation with the aim of restoring intravascular volume, increasing cardiac output, augmenting oxygen delivery and improving tissue oxygenation. Salt and water overload can also result from the administration of large volumes of fluid as drug diluents, artificial nutrition and maintenance fluids. The capillary leak that is inherent to sepsis promotes the extravasation of large amounts of fluid, inducing relative central hypovolemia that often requires further fluid administration, despite interstitial oedema. Capillary leak represents the maladaptive, often excessive, and undesirable loss of fluid and electrolytes with or without protein into the interstitium that generates anasarca and end-organ oedema causing organ dysfunction and eventually failure [9]. Fluid overload should be avoided in this setting.

### Fluid overload

As often described in paediatric populations, the percentage of fluid accumulation is calculated by dividing the cumulative fluid balance in litres by the patient’s baseline body weight and multiplying by 100%. Fluid overload at any stage is defined by a cut-off value of 10% of fluid accumulation, as this is associated with worse outcomes [14, 76, 88].

**Fig. 1:**
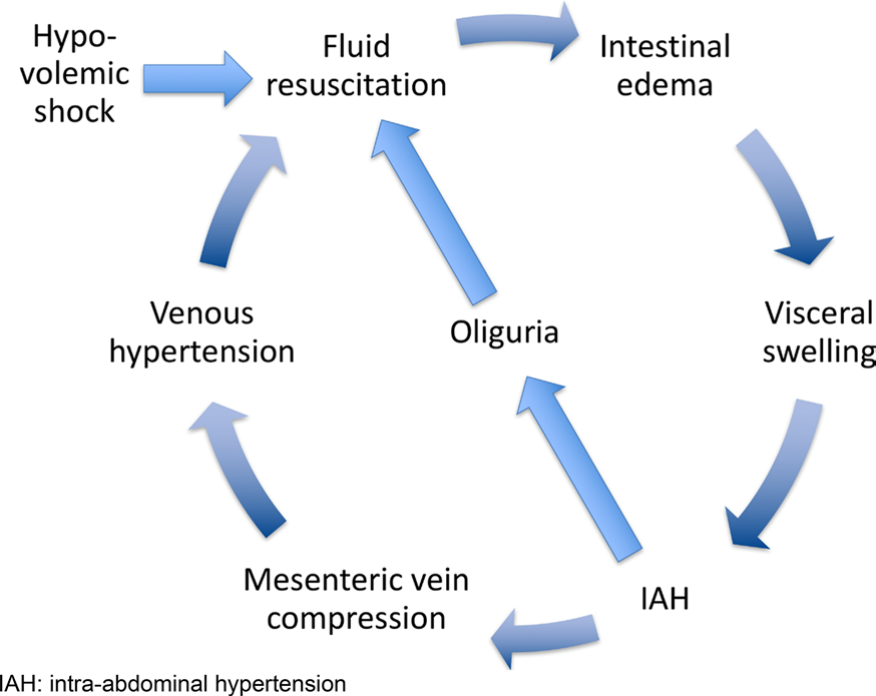
The vicious cycle of septic shock resuscitation. Adapted from Peeters et al. with permission [96]. IAH: intra-abdominal hypertension

**Fig. 2:**
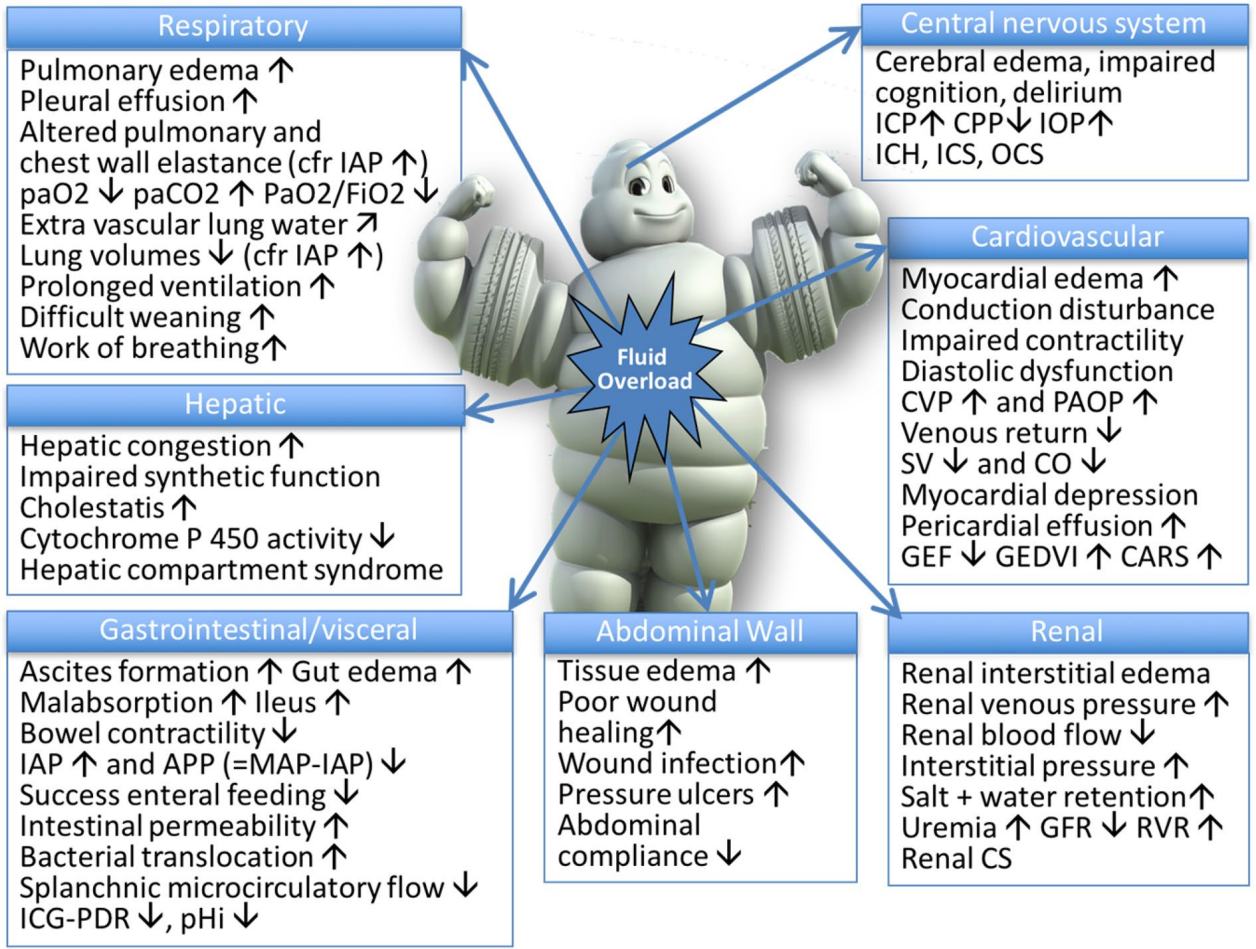
Potential consequences of fluid overload on end-organ function. Adapted from Malbrain et al. with permission [1, 2]. APP: abdominal perfusion pressure, IAP: intra-abdominal pressure, IAH: intra-abdominal hypertension, ACS: abdominal compartment syndrome, CARS: cardio-abdominal-renal syndrome, CO: cardiac output, CPP: cerebral perfusion pressure, CS: compartment syndrome, CVP: central venous pressure, GEDVI: global enddiastolic volume index, GEF: global ejection fraction, GFR; glomerular filtration rate, ICG-PDR: indocyaninegreen plasma disappearance rate, ICH: intracranial hypertension, ICP: intracranial pressure, ICS: intracranial compartment syndrome, IOP: intra-ocular pressure, MAP: mean arterial pressure, OCS: ocular compartment syndrome, PAOP: pulmonary artery occlusion pressure, pHi: gastric tonometry, RVR: renal vascular resistance, SV: stroke volume

Studies demonstrate an association between fluid overload, illustrated by the increase in the cumulative fluid balance, with worse patient centred outcomes [1] in critically ill patients with septic shock [10, 11] and/or acute respiratory distress syndrome [12]. Fluid administration potentially induces a vicious cycle, where interstitial oedema induces organ dysfunction that contributes to fluid accumulation (Fig. 1). Peripheral and generalized oedema is not only of cosmetic concern, as believed by some [13], but harmful to the patient as a whole as it can cause organ oedema and dysfunction [1, 14]. Figure 2 details all the potential harmful consequences of fluid overload on different end-organ systems, with consequential effects on patient morbidity and mortality. As such, fluid therapy can be considered a double-edged sword [1, 15].

Therefore, current treatment of septic shock should include every effort to reduce the cumulative fluid balance. We must always bear in mind that fluids are drugs and oedema is akin to a drug overdose. Their characteristics, indications and contraindications should be carefully considered when choosing their type, their dose, the timing of their administration and the timing for their removal. In parallel, a reasoned fluid strategy requires that we do not consider septic shock as a single “one size fits all” disease, but rather that it is made of different phases, each implying a different therapeutic attitude [16].

## The four D’s of fluid therapy

When prescribing fluids in patients with septic shock, we must take into account their composition and their pharmocodynamic and pharmacokinetic properties. In practice, we should consider the “four D’s” of fluid therapy: drug, dosing, duration and de-escalation (Table 1) [5]. Many clinicians already use these four D’s for the prescription of antibiotics (Table 1).

**Table 1:** Analogy between the four D’s of antibiotic and fluid therapy. Adapted from Malbrain et al. with permission [5]

Description	Terminology	Antibiotics	Fluids
Drug	Inappropriate therapy	More organ failure, longer ICU LOS, longer hospital LOS, longer MV	Hyperchloremic metabolic acidosis, more AKI, more RRT, increased mortality
	Appropriate therapy	Key factor in empiric AB selection is consideration of patient risk factors (e.g. prior AB, duration MV, corticosteroids, recent hospitalization, residence in nursing home)	Key factor in empiric fluid therapy is consideration of patient risk factors (e.g. fluid balance, fluid overload, capillary leak, kidney and other organ function). Do not use glucose as resuscitation fluid
	Combination therapy	Possible benefits: e.g. broader spectrum, synergy, avoidance of emergency of resistance, less toxicity	Possible benefits: e.g. specific fluids for different indications (replacement vs. maintenance vs. resuscitation), less toxicity
	Class	Broad-spectrum or specific, beta-lactam or glycopeptide, additional compounds as tazobactam. The choice has a real impact on efficacy and toxicity	Hypo- or hypertonic, high or low chloride and sodium level, lactate or bicarbonate buffer, glucose containing or not. This will impact directly acid–base equilibrium, cellular hydration and electrolyte regulation
	Appropriate timing	Survival decreases with 7% per hour delay. Needs discipline and practical organization	In refractory shock, the longer the delay, the more microcirculatory hypoperfusion
Dosing	Pharmacokinetics	Depends on distribution volume, clearance (kidney and liver function), albumin level, tissue penetration	Depends on type of fluid: glucose 10%, crystalloids 25%, versus colloids 100% IV after 1 h, distribution volume, osmolality, oncoticity, kidney function
	Pharmacodynamics	Reflected by the minimal inhibitory concentration. Reflected by “kill” characteristics, time (*T* > MIC) versus concentration (Cmax/MIC) dependent	Depends on type of fluid and desired location: IV (resuscitation), IS versus IC (cellular dehydration)
	Toxicity	Some ABs are toxic to kidneys, advice on dose adjustment needed. However, not getting infection under control is not helping the kidney either	Some fluids (HES) are toxic for the kidneys. However, not getting shock under control is not helping the kidney either
Duration	Appropriate duration	No strong evidence but trend towards shorter duration. Do not use AB to treat fever, CRP or chest X-ray infiltrates but use AB to treat infections	No strong evidence but trend towards shorter duration. Do not use fluids to treat low CVP, MAP or UO, but use fluids to treat shock
	Treat to response	Stop AB when signs and symptoms of active infection resolves. Future role for biomarkers (PCT)	Fluids can be stopped when shock is resolved (normal lactate). Future role for biomarkers (NGAL, cystatin C, citrullin, L-FABP)
De-escalation	Monitoring	Take cultures first and have the guts to change a winning team	After stabilization with EAFM (normal PPV, normal CO, normal lactate) stop ongoing resuscitation and move to LCFM and LGFR (= de-resuscitation)

*AB* antibiotic, *AKI* acute kidney injury, *Cmax* maximal peak concentration, *CO* cardiac output, *CRP* C reactive protein, *CVP* central venous pressure, *EAFM* early adequate fluid management, *EGDT* early goal-directed therapy, *IC* intracellular, *ICU* intensive care unit, *IS* interstitial, *IV* intravascular, *LCFM* late conservative fluid management, *L-FABP* L-type fatty acid binding protein, *LGFR* late goal-directed fluid removal, *LOS* length of stay, *MAP* mean arterial pressure, *MIC* mean inhibitory concentration, *MV* mechanical ventilation, *NGAL* neutrophil gelatinase-associated lipocalin, *PCT* procalcitonin, *PPV* pulse pressure variation, *RRT* renal replacement therapy, *UO* urine output

### Drug

We should consider the different compounds: crystalloids versus colloids, synthetic versus blood derived, balanced versus unbalanced, intravenous versus oral. The osmolality, tonicity, pH, electrolyte composition (chloride, sodium, potassium, etc.) and levels of other metabolically active compounds (lactate, acetate, malate, etc.) are all equally important. Clinical factors (underlying conditions, kidney or liver failure, presence of capillary leak, acid–base equilibrium, albumin levels, fluid balance, etc.) must all be taken into account when choosing the type and amount of fluid for a given patient at a given time. Moreover, the type of fluid is different depending on the reason why they are administered. There are only four indications for fluid administration, namely resuscitation, maintenance, replacement and nutrition, or a combination.

#### Resuscitation fluids

Resuscitation fluids are given to correct an intravascular volume deficit in the case of absolute or relative hypovolemia. In theory, the choice between colloids and crystalloids should take into account the revised Starling equation and the glycocalyx model of transvascular fluid exchange [17]. When capillary pressure (or transendothelial pressure difference) is low, as in hypovolemia or sepsis and especially septic shock, or during hypotension (after induction and anaesthesia), albumin or plasma substitutes have no advantage over crystalloid infusions, since they all remain intravascular. However, the glycocalyx layer is a fragile structure and is disrupted by surgical trauma-induced systemic inflammation or sepsis, but also by rapid infusion of fluids (especially saline). Under these circumstances, transcapillary flow (albumin leakage and risk of tissue oedema) is increased, as is the risk to evolve to a state of global increased permeability syndrome (GIPS) [17].

#### Global increased permeability syndrome

Some patients will not transgress to the “flow” phase spontaneously and will remain in a persistent state of *global increased permeability syndrome* and ongoing fluid accumulation [9]. The global increased permeability syndrome can hence be defined as fluid overload in combination with new onset organ failure. This is referred to as “the third hit of shock” [41].

Because of their potential risk, *hydroxyethyl starches* are contraindicated in case of septic shock, burns, patients with acute or chronic kidney injury or in case of oliguria not responsive to fluids (within 6 h) [18]. In other circumstances (post-operative phase, trauma and haemorrhagic shock), starches may still be able to be used as resuscitation fluids, although this remains controversial. Recently, the Coordination Group for Mutual Recognition and Decentralised Procedures-Human (CMDh) has endorsed the European Medicine’s Agency PRAC (Pharmacovigilance Risk Assessment Committee) recommendation to suspend the marketing authorisations of hydroxyethyl starch solutions for infusion across the European Union. This suspension is due to the fact that hydroxyethyl starch solutions have continued to be used in critically ill patients and patients with sepsis, despite the introduction in 2013 of restrictions on use in these patient populations in order to reduce the risk of kidney injury and death (http://www.ema.europa.eu).

#### Septic shock phases

Septic shock starts with an *ebb phase*, which refers to the phase when the patient shows hyperdynamic shock with decreased systemic vascular resistance due to vasodilation, increased capillary permeability, and severe absolute or relative intravascular hypovolemia. The Surviving Sepsis Campaign guidelines mandate the administration of IV fluids at a dose of 30 mL/kg given within the first 3 h, as a possible life-saving procedure in this phase, although there is no randomized controlled trial to support this statement [18]. The *flow phase* refers to the phase after initial stabilization where the patient will mobilize the excess fluid spontaneously. A classic example is when a patient enters a polyuric phase recovering from acute kidney injury. In this post-shock phase, the metabolic turnover is increased, the innate immune system is activated, and a hepatic acute-phase response is induced. This hypercatabolic metabolic state is characterized by an increase in oxygen consumption and energy expenditure [95].

It is justified to use *albumin* as a resuscitation fluid in patients with hypoalbuminemia [18, 19]. *Glucose* should never be used in resuscitation fluid. Surprisingly, *normal saline,* which does not contain potassium, will result in a higher increase in potassium levels in patients with renal impairment compared to a balanced solution (lactated Ringer’s) containing 5 mmol/L of potassium, owing to concomitant metabolic acidosis due to a decreased strong ion difference (SID) [20, 21].

(Ab)normal saline as resuscitation fluid should not be administered in large amounts as it carries the risk of hypernatremic hyperchloremic metabolic acidosis, acute kidney injury and death. The use of *balanced solutions* may avoid these complications. Recent evidence shows the association between fluid-induced chloride loading/hyperchloremia and worse outcomes, probably due to an impact on renal function [22, 23]. In a recent clinical study in human volunteers, a reduction in iatrogenic chloride loading was associated with a decreased incidence of acute kidney injury [24]. Nevertheless, the SALT trial found no significant difference between both types of fluid [25]. Similarly, the recent SPLIT trial also failed to demonstrate a significant difference between saline and a balanced solution (Plasma-Lyte) in critically ill patients [26], although this study has been subject to a lot of criticisms [21]. Recently, as follow-up on the SALT trial, the same authors published the SMART study results [25, 27]. In this pragmatic, cluster-randomized, multiple-crossover trial, the authors assigned 15,802 adults to receive saline (0.9% sodium chloride) or balanced crystalloids (lactated Ringer’s solution or Plasma-Lyte A) and they demonstrated that the use of balanced crystalloids resulted in a lower rate of the composite outcome of death from any cause, new renal replacement therapy, or persistent renal dysfunction than the use of saline [27]. In a companion study at the same institution, noncritically ill adults treated with intravenous fluids in the emergency department had similar numbers of hospital-free days between treatment with balanced crystalloids and treatment with saline [28]. However, similar to the SMART trial, administration of balanced crystalloids resulted in less composite death, new renal replacement therapy or persistent renal dysfunction.

**Fig. 3:**
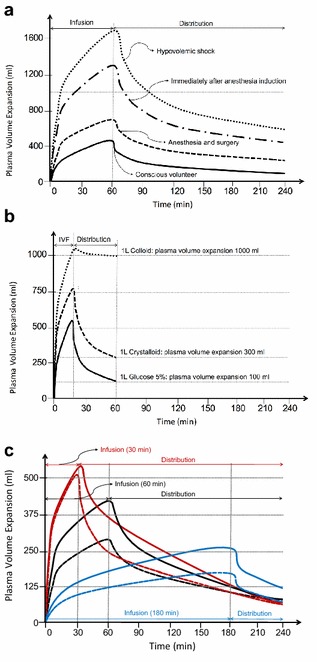
Pharmacokinetics and pharmacodynamics fluids. Original artwork based on the work of Hahn R [29, 43]. **a** Volume kinetic simulation. Expansion of plasma volume (in mL) after intravenous infusion of 2 L of Ringer’s acetate over 60 min in an adult patient (average weight 80 kg), depending on normal condition as conscious volunteer (solid line), during anaesthesia and surgery (dashed line), immediately after induction of anaesthesia due to vasoplegia and hypotension with decrease in arterial pressure to 85% of baseline, (mixed line) and after bleeding during haemorrhagic shock with mean arterial pressure below 50 mmHg (dotted line) (see text for explanation). **b** Volume kinetic simulation. Expansion of plasma volume (in mL) is 100, 300 and 1000 mL, respectively, after 60 min following intravenous infusion of 1 L of glucose 5% over 20 min in an adult patient (solid line), versus 1 L of crystalloid (dashed line), versus 1 L of colloid (dotted line) (see text for explanation). **c** Volume kinetic simulation. Expansion of plasma volume (in mL) after intravenous infusion of 500 mL of hydroxyethyl starch 130/0.4 (Volulyte, solid line) versus 1 L of Ringer’s acetate (dashed line) when administered in an adult patient (average weight 80 kg), over 30 min (red) versus 60 min (black), versus 180 min (blue). When administered rapidly and as long as infusion is ongoing, the volume expansion kinetics are similar between crystalloids and colloids, especially in case of shock, after induction and anaesthesia and during surgery (see text for explanation)

The context-sensitive half-time of crystalloids and colloids may change and vary over time depending on the patient’s condition (Fig. 3). In fact, as long as crystalloids or colloids are infused, they will exert a similar volume expansion effect and their *distribution* and/or *elimination* and excretion will be slowed in case of shock, hypotension, sedation or general anaesthesia [29, 30]. This may explain why crystalloids have a much better short-term effect on the plasma volume than previously believed. Their efficiency (i.e. the plasma volume expansion divided by the infused volume) is 50–80% as long as *infusion* continues and even increases to 100% when the arterial pressure has dropped. Elimination is very slow during surgery and amounts to only 10% of that recorded in conscious volunteers. *Capillary refill* further reduces the need for crystalloid fluid when bleeding occurs. These four factors (distribution–elimination–infusion–capillary refill) limit the need for large volumes of crystalloid fluid during surgery [30].

#### Maintenance fluids

Maintenance fluids are given, specifically, to cover the patient’s daily basal requirements of water, glucose and electrolytes. As such, they are intended to cover daily needs. The basic daily needs are water, in an amount of 25–30 mL/kg of body weight, 1 mmol/kg potassium, 1–1.5 mmol/kg sodium per day and glucose or dextrose 5 or 10% 1.4–1.6 g/kg (to avoid starvation ketosis) [31].

Some specific maintenance solutions are commercially available, but they are far from ideal. There is a lot of debate whether isotonic or hypotonic maintenance solutions should be used. Data in children showed that hypotonic solutions carry the risk of hyponatremia and neurologic complications [32, 33]. However, studies in adults are scarce and indicate that administration of isotonic solutions will result in a more positive fluid balance as compared to hypotonic solutions [34]. This was confirmed in a recent pilot study in healthy volunteers showing that isotonic solutions caused lower urine output, characterized by decreased aldosterone concentrations indicating (unintentional) volume expansion, than hypotonic solutions. Despite their lower sodium and potassium content, hypotonic fluids were not associated with hyponatremia or hypokalemia [24].

#### Replacement fluids

Replacement fluids are administered to correct fluid deficits that cannot be compensated by oral intake. Such fluid deficits have a number of potential origins, like drains or stomata, fistulas, hyperthermia, open wounds, polyuria (salt-wasting nephropathy, cerebral salt wasting, osmotic diuresis or diabetes insipidus) [4].

Data on replacement fluids are also scarce. Several recent guidelines advise matching the amount and composition of fluid and electrolytes as closely as possible to the fluid that is being or has been lost [35, 36]. An overview of the composition of the different body fluids can be found in the NICE guidelines [35]. Replacement fluids are usually isotonic balanced solutions. In patients with fluid deficit due to a loss of chloride-rich gastric fluid, high-chloride solutions, like saline (0.9% NaCl), might be used as replacement fluid.

#### Nutrition fluids

Often overlooked, it is about time to consider parenteral nutrition as another source of intravenous fluids that may contribute to fluid overload. Likewise, nutritional therapy in the critically ill should be seen as “medication” helping the healing process. As such, we might consider also the four D’s of nutritional therapy in analogy to how we deal with antibiotics and fluids [5]: drug (type of feeding), dose (caloric and protein load), duration (when and how long) and de-escalation (stop enteral nutrition and/or parenteral nutrition when oral intake improves) [37].

#### Combination of fluids

A combination of different types of fluids is often justified. For example, numerous combinations may be used in daily practice with regard to resuscitation fluids: blood and crystalloids (trauma), crystalloids early (post-operative hypovolemia), albumin late (sepsis). Similarly, maintenance fluids are often a combination of enteral and parenteral nutrition, other glucose-containing solutions, saline and/or balanced crystalloids to dissolve medications.

### Duration

**Fig. 4:**
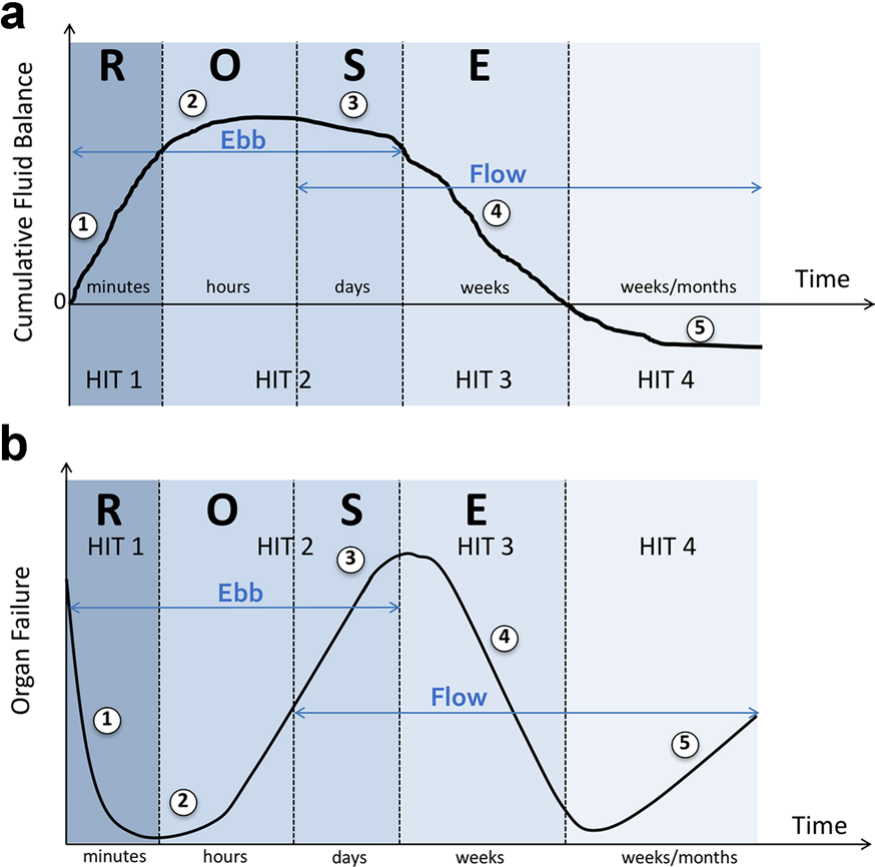
Impact on outcome of appropriate timing of fluid administration. Bar graph showing outcome (mortality %) in different fluid management categories. Comparison of the data obtained from different studies: hospital mortality in 212 patients with septic shock and acute lung injury, adapted from Murphy et al. (light blue bars) [38], hospital mortality in 180 patients with sepsis, capillary leak and fluid overload, adapted and combined from two papers by Cordemans et al. (middle blue bars) [40, 41], 90-day mortality in 151 adult patients with septic shock randomized to restrictive versus standard fluid therapy (CLASSIC trial), adapted from Hjortrup et al. (dark blue bars) [39]. See text for explanation. EA: early adequate fluid management, defined as fluid intake > 50 mL/kg/first 12–24 h of ICU stay. EC: early conservative fluid management, defined as fluid intake < 25 mL/kg/first 12–24 h of ICU stay. LC: late conservative fluid management, defined as 2 negative consecutive daily fluid balances within first week of ICU stay. LL: late liberal fluid management, defined as the absence of 2 consecutive negative daily fluid balances within first week of ICU stay

The longer the delay in fluid administration, the more microcirculatory hypoperfusion and subsequent organ damage related to ischaemia–reperfusion injury. In patients with sepsis [38], Murphy and colleagues compared outcomes related to early adequate versus early conservative and late conservative versus late liberal fluid administration and found that the combination of early adequate and late conservative fluid management carried the best prognosis [38] (Fig. 4). Combined data from other studies confirm that late conservative is maybe more important than early adequate fluid therapy [39–41].

### Dosing

As Paracelsus nicely stated: “All things are poison, and nothing is without poison; only the dose permits something not to be poisonous” Like other drugs, it is the dose of fluids that make them poisonous. As stated before, the risk of excessive fluid overload is well established.

Similar to other drugs, choosing the right dose implies that we take into account the pharmacokinetics and pharmacodynamics of intravenous fluids (Table 1). *Pharmacokinetics* describes how the body affects a drug resulting in a particular plasma and effect site concentration [42]. Pharmacokinetics of intravenous fluids depends on distribution volume, osmolality, tonicity, oncoticity and kidney function. Eventually, the half-time depends on the type of fluid, but also on the patient’s condition and the clinical context (Table 2). When administering 1 L of fluid only, 10% of glucose solution, versus 25–30% of an isotonic crystalloid solution, versus 100% of a colloid solution will remain intravascularly after 1 h, but as stated above the half-life is dependent on other conditions (like infection, inflammation, sedation, surgery, anaesthesia, blood pressure) (Fig. 3) [29, 43].

**Table 2:** Overview of half-life (T1/2) of Ringer’s, glucose and colloid solutions as reported in different studies. Adapted from Hahn R [43]

Category	Study population	*n*	Fluid studied	*T*1/2 (min)
Volunteers	Healthy adults	24	Glucose 2.5%	19
	Healthy adults	9	Glucose 5%	13
	Healthy adults	6	Ringer’s acetate	22–46
	Healthy adults	8	dextran 70	175
	Healthy adults	15	Plasma	197
	Healthy adults	15	Albumin 5%	110
	Healthy adults	20	HES 130/0.4	110
	Dehydrated adults	20	Ringer’s acetate	76
	Healthy children	14	Ringer’s lactate	30
Pregnancy	Normal	8	Ringer’s acetate	71
	Pre-eclampsia	8	Ringer’s acetate	12
	Before caesarean section	10	Ringer’s acetate	175
Surgery	Before surgery	29	Ringer’s acetate	23
	Before surgery	15	Ringer’s lactate	169
	Thyroid	29	Ringer’s acetate	327–345
	Laparoscopic cholecystectomy	12	Glucose 2.5%	492
	Laparoscopic cholecystectomy	12	Ringer’s acetate	268
	Gynaecological laparoscopy	20	Ringer’s lactate	346
	Open abdominal	10	Ringer’s lactate	172
	After hysterectomy	15	Glucose 2.5%	14
	After laparoscopy	20	Ringer’s lactate	17

*HES* hydroxyethyl starch

Volume kinetics is an adaptation of pharmacokinetic theory that makes it possible to analyse and simulate the distribution and elimination of infusion fluids [29]. Applying this concept, it is possible, by simulation, to determine the infusion rate that is required to reach a predetermined plasma volume expansion. Volume kinetics may also allow the quantification of changes in the distribution and elimination of fluids (and calculation of the half-life) that result from stress, hypovolemia, anaesthesia and surgery [43].

*Pharmacodynamics* relates the drug concentrations to its specific effect. For fluids, the Frank–Starling relationship between cardiac output and cardiac preload is the equivalent of the dose effect curve for standard medications. Because of the shape of the Frank–Starling relationship, the response of cardiac output to the fluid-induced increase in cardiac preload is not constant [44]. The effective dose 50 (ED50), in pharmacology, is the dose or amount of drug that produces a therapeutic response or desired effect in 50% of the subjects receiving it, whereas lethal dose 50 (LD50) will result in death of 50% of recipients. Translated to IV fluids, this would be the dose of fluid that induces, respectively, a therapeutic response or death in 50% of the patients. The problem is that the therapeutic response varies from one patient to another. Fluid administration can be toxic (or even lethal) at a high enough dose, as demonstrated in 2007 when a California woman died of water intoxication (and hyponatremia) in a contest organized by a radio station (http://articles.latimes.com/2007/jan/14/local/me-water14). The difference between toxicity and efficacy is dependent upon the particular patient and the specific condition of that patient, although the amount of fluids administered by a physician should fall into the predetermined therapeutic window. Unanswered questions remain: what is an effective dose of IV fluids? What is the exact desired therapeutic effect? What is the therapeutic window? In some patients, volume expansion increases the mean systemic filling pressure (the backward pressure of venous return), but it increases the right atrial pressure (the forward pressure of venous return) to the same extent, such that venous return and, hence, cardiac output do not increase [45]. Hence, venous congestion and backward failure may even play a more important and currently underestimated role [46]. The probability of the heart to “respond” to fluid by a significant increase in cardiac preload varies along the shock time course, and thus, pharmacodynamics of fluids must be regularly evaluated. At the very early phase, fluid responsiveness is constant. After the very initial fluid administration, only one half of patients with circulatory failure respond to an increase in cardiac output [47].

### Fluid responsiveness

Fluid responsiveness indicates a condition in which a patient will respond to fluid administration by a significant increase in stroke volume and/or cardiac output or their surrogates. A *threshold of 15%* is most often used for this definition, as it is the least significant change of measurements of the techniques that are often used to estimate cardiac output [80, 91]. Physiologically, fluid responsiveness means that cardiac output depends on cardiac preload, i.e. the slope of the Frank–Starling relationship is steep. Many studies have shown that fluid responsiveness, which is a normal physiologic condition, exists in only half of the patients receiving a fluid challenge in intensive care units [47].

The adverse effects of fluids must also be considered in their pharmacodynamics. Depending on the degree of vascular permeability, the oedema resulting from fluid administration is highly variable. At the maximum, disruption of the capillary barrier leads to global increased permeability syndrome (GIPS). This pharmacodynamic aspect is also very important in patients with acute respiratory distress syndrome (ARDS), as the effect of a given amount of fluid on the lung function basically depends on the pulmonary vascular permeability [48]. Therefore, even two litres of saline may induce severe respiratory deterioration in a patient with severe ARDS.

### De-escalation

As we will discuss below, the final step in fluid therapy is to consider withholding or withdrawing resuscitation fluids when they are no longer required [1, 14, 15].

Like for antibiotics (Table 1), the duration of fluid therapy must be as short as possible, and the volume must be tapered when shock is resolved. However, many clinicians use certain triggers to start, but are unaware of triggers to stop fluid resuscitation, increasing the potential for fluid overload. As with duration of antibiotics, although there is no strong evidence, there is a trend towards shorter duration of intravenous fluids [39].

## The four phases of fluid therapy

**Fig. 5:**
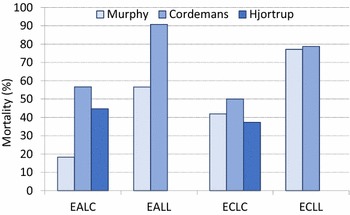
The different fluid phases during shock. Adapted from Malbrain et al. with permission [1]. **a** Graph showing the four-hit model of shock with ebb and flow phases and evolution of patients’ cumulative fluid volume status over time during the five distinct phases of resuscitation: resuscitation (1), optimization (2), stabilization (3) and evacuation (4) (ROSE), followed by a possible risk of Hypoperfusion (5) in case of too aggressive de-resuscitation. See text for explanation. **b** Graph illustrating the four-hit model of shock corresponding to the impact on end-organ function in relation to the fluid status. On admission patients are hypovolemic (1), followed by normovolemia (2) after fluid resuscitation, and fluid overload (3), again followed by a phase going to normovolemia with de-resuscitation (4) and hypovolemia with risk of hypoperfusion (5). In case of hypovolemia (phases 1 and 5), O_2_ cannot get into the tissues because of convective problems, in case of hypervolemia (phase 3) O_2_ cannot get into the tissue because of diffusion problems related to interstitial and pulmonary oedema, gut oedema (ileus and abdominal hypertension). See text for explanation

Not only are the characteristics of fluids important, but also the strategy for their administration. This strategy fundamentally changes along with the time course of septic shock. Recently a three-hit, or even four-hit model of septic shock was suggested trying to answer four basic questions, in which we can recognize four distinct dynamic phases of fluid therapy [40]: resuscitation, optimization, stabilization and evacuation (de-resuscitation) (the acronym ROSE) (Table 3, Fig. 5). The four questions that will be discussed in the next section are “When to start intravenous fluids?”, “When to stop intravenous fluids?”, “When to start de-resuscitation or active fluid removal?” and finally “When to stop de-resuscitation?”

**Table 3:** The ROSE concept avoiding fluid overload. Adapted from Malbrain et al. with permission [1]

	Resuscitation	Optimization	Stabilization	Evacuation
Hit sequence	First hit	Second hit	Second hit	Third hit
Time frame	Minutes	Hours	Days	Days to weeks
Underlying mechanism	Inflammatory insult	Ischaemia and reperfusion	Ischaemia and reperfusion	Global increased permeability syndrome
Clinical presentation	Severe shock	Unstable shock	Absence of shock or threat of shock	Recovery from shock, possible global increased permeability syndrome
Goal	Early adequate goal-directed fluid management	Focus on organ support and maintaining tissue perfusion	Late conservative fluid management	Late goal-directed fluid removal (de-resuscitation)
Fluid therapy	Early administration with fluid boluses, guided by indices of fluid responsiveness	Fluid boluses guided by fluid responsiveness indices and indices of the risk of fluid administration	Only for normal maintenance and replacement	Reversal of the positive fluid balance, either spontaneous or active
Fluid balance	Positive	Neutral	Neutral to negative	Negative
Primary result of treatment	Salvage or patient rescue	Organ rescue	Organ support (homeostasis)	Organ recovery
Main risk	Insufficient resuscitation	Insufficient resuscitation and fluid overload (e.g. pulmonary oedema, intra-abdominal hypertension)	Fluid overload (e.g. pulmonary oedema, intra-abdominal hypertension)	Excessive fluid removal, possibly inducing hypotension, hypoperfusion, and a “fourth hit”

### First phase: Resuscitation

After the *first hit* which can be sepsis, but also burns, pancreatitis or trauma, the patient will enter the “ebb” phase of shock. This life-threatening phase of severe circulatory shock can occur within minutes and is characterized by a strong vasodilation leading to a low mean arterial pressure and microcirculatory impairment (Table 3). It may be accompanied by high (hyperdynamic circulatory shock as seen in sepsis, burns, severe acute pancreatitis, liver cirrhosis, thiamine deficiency, etc.) or low cardiac output (e.g. septic shock with severe hypovolemia or septic shock with sepsis-induced cardiomyopathy).

At this initial phase, usually during the first 3–6 h after the initiation of therapy, fluid resuscitation is commonly administered according to an *early, adequate, goal*-*directed, fluid management* strategy. The modalities of fluid administration at this early phase have been a matter of great debate. In the study by Rivers et al. [49], a protocol-based fluid management called early goal-directed therapy (EGDT) was associated with a significant reduction in mortality compared to standard care. Since this publication, similar outcome benefits have been reported in over 70 observational and randomized controlled studies comprising over 70,000 patients [50]. As a result, EGDT was incorporated as a “resuscitation bundle” into the first 6 h of sepsis management adopted by the Surviving Sepsis Campaign. As such, it has been disseminated internationally as the standard of care for early sepsis management. Recently, a trio of trials (ProCESS [51], ARISE [52] and ProMISe [53]), while reporting an all-time low sepsis mortality, showed no improvement in outcomes with EGDT, questioning the need and pointing towards the potential dangers of protocolized care for patients with severe and septic shock [54, 55]. A recent study employing a combined Bayesian and frequentist methodological approach to evaluate 12 randomized trials and 31 observational studies found that EGDT was potentially harmful in the patients with the highest disease severity [56]. In addition, although conducted in sub-Saharan Africa, three recent trials have demonstrated worse outcomes when administering fluid boluses for resuscitation in patients with septic shock [57–59]. What remains from the EGDT debate is that the rapidity of fluid administration and of the achievement of hemodynamic goals for initial resuscitation is important, even though this aspect has also recently been called into question [60].

In fact, rather than infusing a predefined given amount of fluid, the goal should be individualized for every patient, based on the evaluation of the need for fluids and on the patient’s premorbid conditions [16, 55, 61–64]. In this phase, on an individual basis for each patient, we try to find an answer to the first question: “When to start fluid therapy?”

At the very initial phase of septic shock, answering the question is easy: fluid administration will significantly increase cardiac output in almost all cases. Nevertheless, after the first boluses of fluid, the likelihood of preload unresponsiveness is high. Therefore, at this stage, fluid administration should be conditioned to the positivity of indices and tests predicting fluid responsiveness. However, it must be noted that the state of responsiveness can only be determined a posteriori (after the intervention with administration of fluid bolus) and when a hemodynamic monitoring device is in place to estimate or calculate cardiac output. Therefore, we advocate the use of specific tests to increase the a priori probability and likelihood for a favourable event/outcome, as fluid administration should be limited to responders.

### Fluid bolus

A fluid bolus is the rapid infusion of fluids over a short period of time. In clinical practice, a fluid bolus is usually given to correct hypovolemia, hypotension, inadequate blood flow or impaired microcirculatory perfusion. The volume of fluid bolus is heterogeneous among clinicians [68, 89], typically 500–1000 mL [68]. The minimal fluid volume that is able to increase the backward pressure of venous return is 4 mL/kg [90].

Several of these tests are available today [44]. Instead of using static markers of cardiac preload, which do not reliably predict fluid responsiveness, one should use dynamic indices to predict fluid responsiveness. The principle of these indices is to observe the effect on cardiac output of changes in cardiac preload, either spontaneously induced during mechanical ventilation or provoked by some manoeuvres. If changes are larger than a given threshold, preload responsiveness is present, and the positive response to fluid is likely. Fluid challenge, which has been described years ago [65], is a reliable test for fluid responsiveness, but, since it requires the irreversible administration of fluid, it contributes to excessive fluid administration. The passive leg raise test, which mimics fluid administration [66], has been extensively studied and is now recommended by the Surviving Sepsis Campaign [18]. Other tests utilize the changes in cardiac preload induced by mechanical ventilation. The respiratory changes of pulse pressure and stroke volume, or of the diameter of the venae cava are limited because they cannot be used in many circumstances in critically ill patients [44]. The end-expiratory occlusion test is easy to perform in patients under mechanical ventilation who can tolerate 15-s respiratory holds [67]. However, a cognitive dissonance exists between the fact that most fluid boluses are given to treat hypotension (in 59% of cases in the FENICE trial), while fluid responsiveness can only be defined post-factum by a change in cardiac output [68]. Furthermore, not all that glitters is gold when it comes to predicting fluid responsiveness and some patients may even exhibit an increase in blood pressure with a concomitant decrease in cardiac output after passive leg raising, while others may show the opposite. This relates to the baseline and changing compliance of the aorta over time [69].

### Prediction of fluid responsiveness

This is a process that consists of predicting before fluid administration whether or not subsequent fluid administration will increase cardiac output. It avoids unnecessary fluid administration and contributes to reducing the cumulative fluid balance. It also allows one to undertake fluid removal as it informs that such removal will not result in a hemodynamic impairment [44]. Prediction of fluid responsiveness is based on dynamic tests and indices, which observe the effect on cardiac output of changes in cardiac preload, either spontaneously induced during mechanical ventilation or provoked by some manoeuvres [44]. The threshold to define fluid responsiveness depends on the change in cardiac preload induced by the test (e.g. 15% for fluid challenge, 10% for the PLR test, 5% for the end-expiratory occlusion test).

### Fluid challenge

A fluid challenge is a dynamic test to assess fluid responsiveness by giving a fluid bolus and simultaneously monitoring the hemodynamic effect (e.g. the evolution of barometric or volumetric preload indices). A fluid challenge is therefore also a fluid bolus, which means that it tests the response to treatment by administering the treatment itself up to the level where the treatment has no longer a response. This is why repeated fluid challenges may potentially lead to fluid overload. Recently, it has been shown that in clinical practice there is a marked variability in how fluid challenge tests are performed [68].

### Second phase: Optimization

The *second hit* occurs within hours and refers to ischaemia and reperfusion (Table 3). At this phase, fluid accumulation reflects the severity of illness and might be considered a “biomarker” for it [70]. The greater the fluid requirement, the sicker the patient and the more likely organ failure (e.g. acute kidney injury) may occur [71, 72].

In this phase, we must try to find an answer to the second question: “When to stop fluid therapy?” avoiding fluid overload. Indices of fluid responsiveness are again of utmost importance, since fluid administration should be stopped when these indices become negative [73]. Second, the clinical context must be taken into account. Obviously, more fluid is needed in septic shock from peritonitis than from pneumonia. Third, the decision to refrain from fluid administration should be based on indices that indicate the risk of excessive fluid administration. The presence of lung impairment is the condition that is most likely to be associated with the worst consequences of fluid overload. To estimate the pulmonary risk of further fluid infusion, one may consider the pulmonary artery occlusion pressure measured with the Swan–Ganz catheter. Nonetheless, this does not take into account the degree of lung permeability, which is a key factor in the mechanisms of pulmonary oedema formation [48]. Extravascular lung water measured by transpulmonary thermodilution, as well as the pulmonary vascular permeability index which is inferred from it, might reflect the pulmonary risk of fluid infusion more directly [40, 48, 74]. Intra-abdominal hypertension is also a potential consequence of too much fluid administration [40]. The intra-abdominal pressure should be cautiously monitored in patients at risk [75].

### Passive leg raising test

This test predicting fluid responsiveness consists of moving a patient from the semi-recumbent position to a position where the legs are lifted at 45° and the trunk is horizontal. The transfer of venous blood from the inferior limbs and the splanchnic compartment towards the cardiac cavities mimics the increase in cardiac preload induced by fluid infusion [66]. In general, the threshold to define fluid responsiveness with the passive leg raising test is a 10% increase in stroke volume and/or cardiac output.

### End-expiratory occlusion test

This is a test of fluid responsiveness that consists of stopping mechanical ventilation at end expiration for 15 s and measuring the resultant changes in cardiac output [92–94]. The test increases cardiac preload by stopping the cyclic impediment of venous return that occurs at each insufflation of the ventilator. An increase in cardiac output above the *threshold of 5%* indicates preload/fluid responsiveness [92–94]. When the test is performed with echocardiography, it is better to add the effects of an end-inspiratory occlusion, because the diagnostic threshold of changes in stroke volume is more compatible with precision of echocardiography [67].

### Third phase: Stabilization

With successful treatment, stabilization should follow the optimization phase (homoeostasis), evolving over the next few days (Table 3). It is distinguished from the prior two by the absence of shock or the imminent threat of shock. As previously described, the focus is now on organ support and this phase reflects the point at which a patient is in a stable steady state [1, 76] (Table 3).

Fluid therapy is now only needed for ongoing maintenance in the setting of normal fluid losses (i.e. renal, gastrointestinal, insensible) and replacement fluids if the patient is experiencing ongoing losses because of unresolved pathologic conditions [1, 76]. Since persistence of a positive daily fluid balance over time is strongly associated with a higher mortality rate in septic patients [11, 77], clinicians should also be aware of the hidden obligatory fluid intake, as it may contribute more than a litre daily [78].

### Fluid balance

Daily fluid balance is the sum of all fluid intakes and outputs over 24 h, and the cumulative fluid balance is the sum of daily fluid balances over a set period of time [76, 87]. Intakes include resuscitation, but also maintenance fluids. Outputs include urine, ultrafiltration fluids, third space or gastrointestinal losses and should ideally also include insensible losses, even though they are difficult to quantify.

Maintenance fluids should be used only to cover daily needs, and their prescription should take these other sources of fluids and electrolytes into account. Therefore, when a patient already receives daily needs of water, glucose and electrolytes via other means (enteral or parenteral nutrition, medication solutions, etc.), specific intravenous maintenance fluids should be stopped.

### Fourth phase: Evacuation

After the second hit, the patient may either further recover, entering the “flow” phase with spontaneous evacuation of the excess fluids that have been administrated previously, or, as is the case in many critically ill patients, the patient remains in a “no-flow” state followed by a *third hit,* usually resulting from global increased permeability syndrome with ongoing fluid accumulation due to capillary leak [17, 79]. In any case, the patient enters a phase of “de-resuscitation” (Table 3). This term was first suggested in 2012 [41] and finally coined in 2014 [1]. It specifically refers to *late goal-directed fluid removal* and *late conservative fluid management.*

*Late goal-directed fluid removal* involves aggressive and active fluid removal using diuretics and renal replacement therapy with net ultrafiltration. It is characterized by the discontinuation of invasive therapies and a transition to a negative fluid balance [40]. *Late conservative fluid management* describes a moderate fluid management strategy following the initial treatment in order to avoid (or reverse) fluid overload. Recent studies showed that two consecutive days of negative fluid balance within the first week of the intensive care unit stay is a strong and independent predictor of survival [1].

In this de-resuscitation phase, we try to find an answer to the third and fourth question: “When to start fluid removal?” and “When to stop fluid removal?” in order to find the balance between the benefits (reduction in second and third space fluid accumulation and tissue oedema) and risk (hypoperfusion) of fluid removal. To answer these questions, testing preload responsiveness may still be useful. Indeed, if no preload responsiveness is detected, it is reasonable to assume that fluid removal will not induce a reduction in cardiac output [80]. On the opposite, positive indices of preload responsiveness might indicate the limit of fluid removal and could even be a target to reach when removing fluids.

Obviously, the risk at this phase is to be too aggressive with fluid removal and to induce hypovolemia, which may trigger a “fourth hit” for hemodynamic deterioration and hypoperfusion (Fig. 5). If fluid is needed at this phase, the use of albumin seems to have positive effects on vessel wall integrity facilitates achieving a negative fluid balance in hypoalbuminemia and may be less likely to cause nephrotoxicity [81].

This four-phase approach should be better characterized by some epidemiological studies. Its prognostic impact might be significant, because it may lead to a reduction in the cumulative fluid balance, which by itself is clearly associated with poor prognosis (Fig. 4). Similar principles have also been suggested by others, confirming the need for a multicenter prospective clinical trial with a biphasic fluid therapy approach, starting with initial early adequate goal-directed treatment followed by late conservative fluid management in those patients not transgressing spontaneously from the ebb to the flow phase [14, 15, 70, 76, 82–86]. The RADAR (Role of Active De-resuscitation After Resuscitation) trial may help to find such answers (http://www.hra.nhs.uk/news/research-summaries/radar-icu/).

## Conclusions

There are only four major *indications* for fluid administration in the critically ill: resuscitation, maintenance, replacement and nutrition (enteral or parenteral). In this review, a conceptual framework is presented looking at fluids as drugs by taking into account the four *D’s* (drug selection, dose, duration and de-escalation) and the four *phases* of fluid therapy within the ROSE concept (resuscitation, optimization, stabilization and evacuation). The four *hits* model is presented herein. This will provide answers to the four basic *questions* surrounding fluid therapy: (1) When to start IV fluids? (2) When to stop fluid administration? (3) When to start fluid removal and finally (4) When to stop fluid removal? In analogy to the way we deal with antibiotics in critically ill patients, it is time for fluid stewardship.

## Abbreviations

EGDT: early goal-directed therapy
GIPS: global increased permeability syndrome
ROSE: acronym for resuscitation–optimization–stabilization–evacuation
